# Supplementary material for: Social network and tourism consumption by households: Evidence from China
Source: PLoS One. 2022 Sep 30;17(9):e0275418. doi: 10.1371/journal.pone.0275418 (PMC9524651; doi:10.1371/journal.pone.0275418)
Supplement: Attachment — Submitted filename: Response to Reviewers.docx [file pone.0275418.s002.docx]

Response to Review Comments

**Response to Reviewer 1 Comments:**

1. Please highlight the key points in the abstract.

Response1: We accept the suggestion of the reviewer. We have updated the abstract section of the article following the structure of purpose, problem statement, methods, results and conclusions, and added specific research findings especially.

2. The introduction should further highlight what is the motivation of the paper.

Response2: We accept the suggestion of the reviewer. We further clarify the motivation of this study in the third paragraph of the introduction.

3. The literature review section is also not focused enough. Please read Effects of rural revitalization on rural tourism. Journal of Hospitality and Tourism Management (2021), https://doi.org/10.1016/j.jhtm.2021.02.008. An input-output analysis of transportation equipment manufacturing industrial transfer: Evidence from Beijing-Tianjin-Hebei region, China. Growth and Change (2021).https://doi.org/10.1111/grow.12571. The influence of high-speed rail on ice–snow tourism in northeastern China. Tourism Management（2020）, doi:10.1016/j.tourman.2019.104070.

Response3: We accept the suggestion of the reviewer. We have updated the literature review section to make it more focused by adding new articles and removing irrelevant articles.

4. It is suggested to update the data of the paper, so that the results may be more meaningful.

Response4: Although the reviewer's suggestion is very reasonable, we are very sorry that the data cannot be updated. The basic data used in this article comes from the 2018 China Family Panel Studies (CFPS) database, including household data and individual data. The latest issue of the 2020 CFPS database has only released individual data and parent-response data of children to the public. Other data sets are still being cleaned and evaluated, and have not yet been released. So, we can’t have access to the latest household data to update the data of the paper.

5. Drawing lessons from the study by Yi et al. How did you draw this lessons?

Response5: In the paper entitled “The Family Social Network and the Rural Household Saving Behavior: a Case Study Based on China’s Villages”, Yi et al. used the logarithm of the amount of gift or money given by households as a surrogate variable for the social network. The reason they gave was that the interaction between families, relatives, friends and other social networks was directly reflected in the receipt and payment of key money. The expenditure of gift was a relatively stable cash flow. The gifts or money given by families to relatives and friends could be regarded as the investment and maintenance of family social network, and it well reflected the three characteristics of social network: the network of relatives and friends. The scale, the closeness of the network of relatives and friends, and the support ability of the network of relatives and friends. So, following Yi et al., we chose “gift of money” as the measurement index of households’ social network.

6. The results should be better described, discussed and justified.

Response6: We accept the suggestion of the reviewer. We have conducted a more detailed analysis and discussion of the results of this paper, and optimized the research conclusions to make them more concise and clearer. In addition, some literature dialogues have been added.

**Response to Reviewer 2 Comments:**

1.the language of the paper should be further improved.

Response1: We accept the suggestion of the reviewer. After modifying it ourselves, we entrust the Bioedit Ltd Company for editing proper English language, grammar, punctuation, spelling, and overall style of this manuscript.

2.why does the authors show? I think it should be moved to the methology part.

Response2: We accept the suggestion of the reviewer. We move Quantile Regression Model in the part 4.4.5 to the part 3.4.2.

3.There is no discussion in the paper, which weaken the theoretical contribution of the research.

Response3: Thanks for the reviewer's suggestion. We have the discussion of the results during the analysis, so there is no separate discussion section.

4.I think implication is a better word than recommendations.

Response4: We accept the suggestion of the reviewer. We change the title “5.2.reommendations” to “5.2. implications”, and improve the content.
